# Dual RNASeq Reveals NTHi-Macrophage Transcriptomic Changes During Intracellular Persistence

**DOI:** 10.3389/fcimb.2021.723481

**Published:** 2021-08-23

**Authors:** Jodie Ackland, Ashley I. Heinson, David W. Cleary, Myron Christodoulides, Tom M. A. Wilkinson, Karl J. Staples

**Affiliations:** ^1^Clinical and Experimental Sciences, Faculty of Medicine, University of Southampton, Southampton, United Kingdom; ^2^NIHR Southampton Biomedical Research Centre, University Hospital Southampton NHS Foundation Trust, Southampton, United Kingdom; ^3^Wessex Investigational Sciences Hub, Southampton General Hospital, Faculty of Medicine, University of Southampton, Southampton, United Kingdom

**Keywords:** macrophage, NTHi, intracellular persistence, dual RNAseq, host-pathogen interactions

## Abstract

Nontypeable *Haemophilus influenzae* (NTHi) is a pathobiont which chronically colonises the airway of individuals with chronic respiratory disease and is associated with poor clinical outcomes. It is unclear how NTHi persists in the airway, however accumulating evidence suggests that NTHi can invade and persist within macrophages. To better understand the mechanisms of NTHi persistence within macrophages, we developed an *in vitro* model of NTHi intracellular persistence using human monocyte-derived macrophages (MDM). Dual RNA Sequencing was used to assess MDM and NTHi transcriptomic regulation occurring simultaneously during NTHi persistence. Analysis of the macrophage response to NTHi identified temporally regulated transcriptomic profiles, with a specific ‘core’ profile displaying conserved expression of genes across time points. Gene list enrichment analysis identified enrichment of immune responses in the core gene set, with KEGG pathway analysis revealing specific enrichment of intracellular immune response pathways. NTHi persistence was facilitated by modulation of bacterial metabolic, stress response and ribosome pathways. Levels of NTHi genes *bioC*, *mepM* and *dps* were differentially expressed by intracellular NTHi compared to planktonic NTHi, indicating that the transcriptomic adaption was distinct between the two different NTHi lifestyles. Overall, this study provides crucial insights into the transcriptomic adaptations facilitating NTHi persistence within macrophages. Targeting these reported pathways with novel therapeutics to reduce NTHi burden in the airway could be an effective treatment strategy given the current antimicrobial resistance crisis and lack of NTHi vaccines.

## Introduction

*Haemophilus influenzae* is a human-restricted pathobiont (Erwin and Smith, 2007) and is commonly isolated from the nasopharynx, middle ear and respiratory tract (King, 2012; Swords, 2012; Ahearn et al., 2017). *H. influenzae* can be divided into typeable and nontypeable strains depending on the presence or absence of a polysaccharide capsule. Encapsulated strains are classified into six serotypes (a-f), with strains not in possession of a capsule unable to be serotyped and are designated as nontypeable *Haemophilus influenzae* (NTHi). NTHi is associated with various diseases including pneumonia, meningitis, sinusitis, otitis media and exacerbations of chronic respiratory diseases such as Chronic Obstructive Pulmonary Disease (COPD) and asthma (King, 2012; Finney et al., 2014; Green et al., 2014; Van Eldere et al., 2014; McCann et al., 2016). Although NTHi is implicated in exacerbations of chronic respiratory diseases, NTHi has also been isolated from the airway during stable periods of disease (Wood et al., 2010; Iikura et al., 2015; Zhang et al., 2016; Wilkinson et al., 2017; Mayhew et al., 2018). The duration of NTHi airway colonisation varies, with longitudinal studies suggesting persistence ranges from months up to as long as 7 years (Murphy et al., 2004; Román et al., 2004; Gallo et al., 2018).

NTHi has traditionally been considered an extracellular pathogen, however an increasing number of reports suggest NTHi is able to invade immune cells to enhance airway persistence and survival (Ahrén et al., 2001; Craig et al., 2001; Craig et al., 2002; King et al., 2008; Morey et al., 2011). The predominant innate immune cell in the healthy respiratory tract is the macrophage, which orchestrates the airway immune defence response, regulates inflammation, maintains homeostasis and participates in immune resolution processes (Mosser and Edwards, 2008; Byrne et al., 2015). Despite these key processes, accumulating evidence suggests that macrophages are a target of infection for NTHi. One of the first indications of NTHi invasion of immune cells was provided by Forsgren et al., who used transmission electron microscopy to visualise replicating, intracellular NTHi within macrophage-like cells isolated from adenoid tissue (Forsgren et al., 1994). Subsequent *in vitro* studies have reported varying mechanisms of NTHi invasion of phagocytic cells including receptor-mediated endocytosis (Ahrén et al., 2001), lipid raft mediated endocytosis (Martí-Lliteras et al., 2009) and phagocytosis (Clementi and Murphy, 2011), with strain-dependent differences between clinical isolates reported (Craig et al., 2001). Despite the prominent role of macrophages in the innate immune response against NTHi, the mechanisms of NTHi intracellular persistence within macrophages are not well understood.

Furthermore, macrophage dysfunction has been demonstrated in chronic respiratory diseases. In asthma, macrophages have an altered phenotype (Staples et al., 2012) and reduced phagocytic activity, which worsens with asthma severity (Liang et al., 2014). Monocyte-derived macrophages from the blood of asthma patients also exhibit impaired phagocytosis, suggesting the phagocytic defect is not limited to the lung (Liang et al., 2014). A similar defect in phagocytic capacity has also been reported in patients with COPD, with reduced uptake of NTHi by both alveolar macrophages and monocyte-derived macrophages (Berenson et al., 2006; Taylor et al., 2010), which is associated with exacerbation frequency (Singh et al., 2021) and disease severity (Berenson et al., 2013). Impairment in macrophage regulation and clearance of NTHi could contribute to persistent and chronic NTHi colonisation of the lung.

Genomic studies have identified NTHi genome evolution during airway persistence (Pettigrew et al., 2018), however RNASeq can offer insights into the dynamic transcriptomic changes which occur during host-pathogen interactions. Previous studies have shown modulation of NTHi gene expression contributes to enhanced intracellular survival within epithelial cells by upregulation of bacterial stress response genes and metabolic pathways (Craig et al., 2002; Baddal et al., 2015). It is not clear whether NTHi similarly modulates gene expression during intracellular persistence of macrophages, enabling NTHi survival and evasion of macrophage immune responses to facilitate chronic airway colonisation. Given the challenges associated with developing an efficient NTHi vaccine, identifying how NTHi can persist within the airway could be crucial in guiding the development of antimicrobial therapeutics aimed at reducing NTHi burden in chronic respiratory disease. Although the initial stages of NTHi invasion and entry into macrophages have been documented (Ahrén et al., 2001; Martí-Lliteras et al., 2009; Clementi and Murphy, 2011), it is not clear how NTHi is able to survive once inside a macrophage. Therefore, the aim of this work was to investigate NTHi-macrophage interactions using dual RNASeq to determine transcriptomic changes during intracellular persistence.

## Materials and Methods

### MDM Culture and Infection

Blood from healthy volunteers was collected in accordance with the protocol as approved by the Hampshire A Research Ethics Committee (13/SC/0416). Monocytes isolated from blood were seeded at 5 x 10^5^ cells per well and differentiated for 12 d as previously described (Staples et al., 2012; Cooper et al., 2018). On day 12, culture media was replaced with reduced serum (0.1% foetal bovine serum, FBS), antibiotic-free RPMI media and either left uninfected or were challenged with NTHi at multiplicity of infection (MOI) 100 for 6 h. At 6 h, media was removed and cells washed twice before addition of reduced serum RPMI supplemented with 500 μg/ml gentamicin to wells for 90 min in order to kill and remove extracellular bacteria (referred to as ‘6 h time point’). Gentamicin-containing RPMI media was removed and cells were washed before further incubation in reduced serum RPMI media until harvest at 24 h (referred to as ‘24 h time point’). Presence of NTHi at this 24 h time point in the absence of antibiotics is regarded as persistence.

### NTHi Culture and Growth

NTHi strains ST14, ST408 and ST201 isolated from COPD patients undergoing bronchoscopy (Osman et al., 2018, Wallington et al., 2018), and NTHi-GFP-375^SR^ (kindly gifted by Dr. Derek Hood, MRC Harwell, UK) (Mulay et al., 2016) were grown as previously described (Kirkham et al., 2013). Briefly, NTHi was cultured from frozen stock on to chocolate blood agar plates (CHOC, Oxoid, Basingstoke, UK) and incubated overnight at 37°C, 5% CO_2_. Single NTHi colony forming units (CFU) were selected and inoculated in supplemented Brain Heart Infusion (BHI) media (30 mg/L Hemin (Sigma-Aldrich, Paisley, UK), 10 mg/L β-Nicotinamide adenine dinucleotide (β-NAD, Sigma) and 44 ml/L glycerol at 37°C, 5% CO_2_ for 8 h to achieve mid-log phase. Heat inactivated FBS (20%) was added to culture and 1 ml aliquots were stored at -80°C until required. Counts of frozen NTHi stocks were routinely performed to determine the concentration of aliquots and to ensure NTHi viability remained stable over time. NTHi aliquots for infection were defrosted and transferred to a fresh tube containing 500 µl PBS and centrifuged at 800 g for 10 min, 4°C to pellet the bacteria. The remaining supernatant was discarded and the pellet was resuspended in reduced serum, antibiotic-free RPMI media and added to MDM. For assessment of gene expression in planktonic state NTHi, NTHi was prepared for infection as above, but the cell pellet was not resuspended in RPMI and added to MDM and instead was treated with TRIzol reagent (Life Technologies).

### Quantifying Intracellular NTHi Persistence

To enumerate NTHi recovered from MDM, MDM were lysed with 1 x PermWash™ (PW, BD Biosciences) for 20 min. Cells were serially diluted and plated on CHOC agar plates (Oxoid) and incubated at 37°C, 5% CO_2_ overnight.

### Visualisation of NTHi Persistence

MDM were challenged with GFP-375^SR^ as described and cells were harvested using Cell Dissociation Solution (Sigma) for 20 min at 37°C. The cell suspension was recovered and centrifuged to generate a cell pellet. The cell pellet was resuspended in PBS and streaked onto PolyFrost Microslides (Solmedia, Shrewsbury, UK). Once dry, slides were fixed using 4% paraformaldehyde (PFA) for 15 min. Excess PFA was removed and slides were washed in PBS and left until completely dried. Once dry, 25 μl Vectashield^®^ Mounting Medium solution containing DAPI nuclear stain (1.5 μg/ml) (Vector Laboratories, Inc. Burlingame, CA) was added to each spot and a glass coverslip mounted on top. Slides were visualised using Axioscope KS400 fluorescence microscope using Carl Zeiss Axioscope 3.0 software.

### Dual RNASeq

RNA extraction for sequenced samples was performed using a miRNeasy kit (QIAGEN^®^, Manchester, UK), including a DNase I (QIAGEN^®^) treatment step, according to the manufacturer’s instructions. RNA quality was assessed using an Agilent Bioanalyzer 2100 system, prior to sequencing of ribosomal RNA-depleted RNA (Illumina^®^ Ribo-Zero Plus rRNA Depletion Kit), which was performed by Novogene (Hong Kong). Libraries were generated using the NEBNext^®^Ultra™ Directional RNA Library Prep Kit for Illumina^®^ (NEB, USA). Library quality was assessed on the Agilent Bioanalyzer 2100 system and quantified using a Qubit 2.0 fluorometer (Life Technologies). Sequencing was performed using NovaSeq 6000 Illumina^®^ platform and 150-base pair (bp) paired end reads were generated using a sequencing depth of 90 million reads. Reads containing adapter sequences, poly-N and low quality reads were removed to obtain clean reads. Mapping of the cleaned, raw data to the respective reference genomes separated MDM and NTHi transcripts *in silico*. The Spliced Transcripts Alignment to a Reference (STAR) (Dobin et al., 2013) software (version 2.5) was used to map reads to the human genome (hg38) and Bowtie2 (Langmead and Salzberg, 2012) (version 2.2.3) was used to map reads to the NTHi ST14 genome. Aligned reads were quantified using HTSeq (Anders et al., 2015) and data were filtered to remove lowly expressed reads prior to differential gene expression analysis. Two packages, edgeR (Robinson et al., 2009) and DESeq2 (Anders and Huber, 2010), were used to determine changes in host gene expression between uninfected or NTHi-challenged MDM at 6 h or 24 h or to determine changes in NTHi gene expression between 6 h and 24 h. Significantly differentially expressed genes (DEGs) were determined as log_2_ FC ± 2 (MDM) or log_2_ FC ± 1 (NTHi) and FDR p-value <0.05 by both edgeR and DESeq2. To explore the biological relevance of the significant DEGs, gene list enrichment analysis and Kyoto Encyclopaedia of Genes and Genomes (KEGG) pathway analysis was performed using ToppFunn (Chen et al., 2009) and ShinyGo (Ge et al., 2020) using default parameter settings (FDR multiple correction method and enrichment significance cut off level 0.05). Clustering of the top gene ontology terms was performed in Cytoscape using the EnrichmentMap (Merico et al., 2010) and AutoAnnotate (Kucera et al., 2016) plugins.

### Strain Comparisons

To assess NTHi strain diversity, the ParSNP package from the Harvest suite (Treangen et al., 2014) was used to analyse 7 clinical isolates of NTHi. The 86-028NP assembly, (GenBank number CP000057.2), was downloaded from https://www.ncbi.nlm.nih.gov/nuccore to be used as a reference. Default parameters were used to construct an assembly-based core-SNP phylogeny. From this analysis, three strains isolated from different anatomical locations and identified on three different clades of the constructed phylogenetic tree were chosen to infect MDM as described for strain comparison infection experiments.

### RNA Isolation and qPCR

Samples were treated with TRIzol reagent (Life Technologies) and RNA was isolated according to manufacturer’s instructions. Reverse transcription to produce cDNA was carried out according to the manufacturer’s instructions using a High Capacity cDNA Reverse Transcription Kit (Life Technologies) with random hexamers. Quantitative PCR (qPCR) was performed using TaqMan universal PCR master mix, with all primers obtained from Applied Biosystems (**Table S3**). The qPCR reactions were performed at 95°C for 10 min and 40 cycles of 95°C for 15 s and 60°C for 1 min using a 7900HT Fast Real-Time PCR System. Gene expression of target genes were normalised either to *B2M* (MDM) or *rho* (NTHi) using the delta-delta Ct method.

### Lactate Dehydrogenase Assay (LDH)

LDH release into culture supernatants was assessed by the CytoTox 96^®^ Non-Radioactive Cytotoxicity Assay according to the manufacturer’s instructions (Promega, Madison, USA). Briefly, 50 μl of harvested supernatant and 50 μl CytoTox 96^®^ Reagent was added to a 96 well plate and incubated for 30 min in the dark at room temperature. 50 μl of stop solution was added and the absorbance read on a microplate reader at 490 nm (Multiskan Ascent, Agilent Technologies, Wokingham, UK). Optical Density (OD) reading of a media only control was regarded as background and subtracted from sample values.

### Quantification of MDM Mediator Release

IL-1β, IL-6 and IL-8 release into cell culture supernatants was assessed by DuoSet ELISA kits, which was carried out according to manufacturer’s instructions (R&D Systems). IL-10, IL-15, IL-17C, IL-36β and TNF-α release into cell culture supernatants was assessed by a customised Luminex Human Magnetic Assay according to manufacturer’s instructions (R&D Systems). Further information is supplied in the **Supplementary Methods**.

### Statistics

Statistical analysis was performed using GraphPad Prism (version 8 GraphPad Software, San Diego, USA) and statistical significance was determined as p<0.05. For paired data, Wilcoxon matched-pairs signed rank test between two groups or Friedman test with Dunn’s *post hoc* test for multiple comparison testing between more than two groups for one independent variable were used. For unpaired data, Kruskal-Wallis with Dunn’s *post hoc* test for multiple comparison testing was used.

## Results

### NTHi Persists Intracellularly Within Macrophages

**Figure 1:**
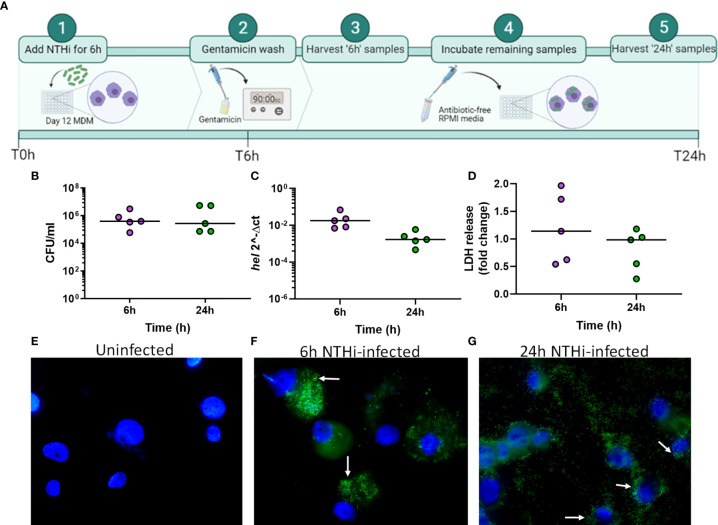
Modelling NTHi infection of macrophages. **(A)** Model workflow: MDM were challenged with NTHi for 6 h, washed with gentamicin for 90 min to remove extracellular NTHi and left to incubate in antibiotic-free media until 24 h (created using BioRender.com). **(B)** The 6 h and 24 h time point MDM samples were lysed and plated to quantify the amount of NTHi associated with MDM. **(C)** RNA was harvested from the 6 h and 24 h uninfected and NTHi-infected MDM samples to assess the presence of NTHi RNA through detection of the *hel* gene by qPCR. Expression of the *hel* gene was normalised to *B2M*. **(D)** MDM viability was not impacted by NTHi ST14 infection, as assessed by LDH release into cell culture supernatants at 6 h and 24 h. **(B–D)** (n = 5) show paired data and lines indicate medians. Data were analysed by Wilcoxon signed-rank test and no statistical significance was determined. GFP-NTHi was used to visually confirm NTHi association with MDM at the 6 h and 24 h time points. Uninfected and NTHi-infected MDM were streaked and fixed onto glass sides followed by staining with DAPI. Slides were visualised using the AxioScope KS400 fluorescence microscope at 100x magnification. **(E)** Uninfected MDM, **(F)** GFP-NTHi infected MDM at the 6 h time point and **(G)** GFP-NTHi infected MDM at the 24 h time point. White arrows indicate NTHi associated with MDM cell nuclei.

To determine NTHi-macrophage transcriptomic changes during intracellular persistence, NTHi persistence was modelled using a monocyte-derived macrophage (MDM) model that has previously been described to resemble alveolar macrophages (Akagawa et al., 2006; Tudhope et al., 2008; Taylor et al., 2010). However, as a challenging and limiting factor of dual RNASeq is that bacterial RNA can make up less than 1% of the total RNA in an infected cell (Marsh et al., 2017), it was first important to determine the time point at which the highest amount of intracellular NTHi was present. As such, a timecourse was first performed to assess the ability of NTHi to reside intracellularly. MDM were challenged with a clinical isolate of NTHi (ST14) for 2 h, 6 h or 24 h followed by a 90 min gentamicin wash to kill and remove extracellular NTHi, resulting in only intracellular NTHi present in the model. Higher levels of NTHi CFU were recovered from MDM after 6 h compared to both 2 h (not significant) and 24 h (p=0.04 - **Figure S1**). As intracellular NTHi presence was highest at 6 h, the infection model was extended following the 90 min gentamicin wash. MDM were incubated in antibiotic-free media until 24 h to assess the ability of the intracellular NTHi to persist (**Figure 1A**).

At both 6 h and 24 h, NTHi was detected and quantified by live viable counting, with no significant difference in NTHi CFU between time points indicating the ability of NTHi to persist until at least 24 h (**Figure 1B**). Although live NTHi was quantified using CFU, it was important to determine whether NTHi RNA could be detected at these recovered amounts, to ensure NTHi RNA was detectable prior to sequencing. The *hel* gene encodes for a conserved NTHi outermembrane protein (lipoprotein e) and has previously been used to assess the presence of *H.influenzae* in clinical samples by PCR (Yadav et al., 2003; Coughtrie et al., 2018). Expression of the NTHi *hel* gene was detected at both 6 h and 24 h by qPCR (**Figure 1C**). Despite continued presence of NTHi, no impact on MDM viability was detectable, as measured by LDH release into cell culture supernatants (**Figure 1D**).

Next, this optimised model was used to visualize NTHi persistence within MDM at 6 h and 24 h using a GFP-NTHi strain (GFP-NTHi-375^SR^). MDM were infected as described and harvested after a gentamicin wash was used to kill and remove all extracellular NTHi. MDM infected with GFP-NTHi had clear evidence of GFP fluorescence at both 6 h and 24 h, which was closely associated with the macrophage nuclei (**Figures 1E–G**). The use of a gentamicin wash indicated that the visualized NTHi resided intracellularly within MDM, which was further confirmed by recovery of live GFP-NTHi by viable counting at 6 h and 24 h (**Figure S2A**). Intracellular GFP-NTHi was further quantified by flow cytometry and no significant difference was observed between the two time points (**Figures S2B, C**).

### Distinct Temporal Transcriptomic Profiles Elicited by Macrophages During Intracellular Persistence

Dual RNASequencing was performed using RNA harvested at the 6 h and 24 h time points from five biological repeats. Mapping to the reference human genome accounted for 93% of total unique reads in uninfected samples (**Figure S3**). For infected samples, a lower median number of unique reads mapped to the human genome (77%), likely due to the presence of NTHi, which accounted for 11% of the total median unique reads. The remaining reads consisted of unmapped or multiple-mapped reads.

**Figure 2:**
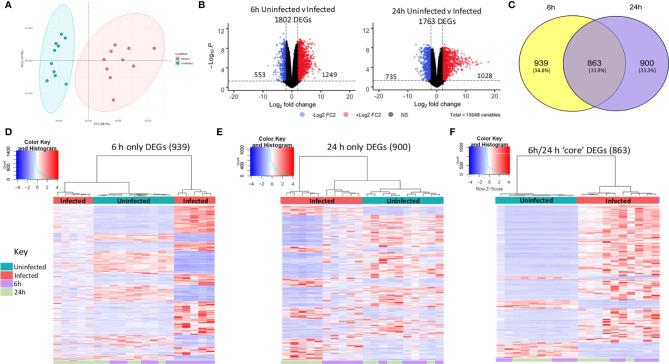
Distinct macrophage transcriptomic profiles in response to NTHi persistence. **(A)** Principal component analysis of the MDM data set identified that samples clustered based on infection status, with uninfected samples in blue and NTHi infected samples in red. **(B)** Differential gene expression analysis found 1802 MDM DEGs at the 6 h time point (left) and 1763 MDM DEGs at 24 h time point (right) (log_2_ FC2 cut off, FDR p < 0.05). **(C)** Venn diagram showing the regulation of MDM DEGs in a time-dependent manner, with 939 genes only differentially expressed at 6 h, 900 genes only differentially expressed at 24 h and 863 genes differentially expressed across both 6 h and 24 h. Heatmap visualisation of the gene expression profiles indicate time-dependent clustering of samples. **(D)** Clustering of samples based on the expression profile of the MDM DEGs at the 6 h time point only show clustering of the 6 h time point sample away from uninfected samples at both time points, as well as the NTHi infected 24 h time point samples. **(E)** Similarly, the NTHi infected samples harvested at the 24 h time point cluster away from all uninfected and 6 h infected samples. **(F)** In contrast, based on the expression of the 863 ‘core’ DEGs, the NTHi-infected samples clustered together separately away from the uninfected samples, with no sub clustering based on time point. Clustering was performed using Euclidean distance and Ward linkage methods. Heatmap colour key indicates sample metadata; blue = uninfected samples, red = infected samples, purple = 6 h time point samples and green = 24 h time point samples.

Exploratory analysis of the macrophage data using principal component analysis (PCA) identified two distinct clusters separated by the first principal component (PC1, 69.3%), which were identified to be either infected or uninfected MDM (**Figure 2A**). Differential gene expression analysis between uninfected and infected MDM identified 1802 differentially expressed genes (DEGs) at 6 h and 1763 DEGs at 24 h (**Figure 2B**, log_2_ FC ± 2, FDR p<0.05). This represents 11.9% and 11.7% of the starting number of MDM genes (15048) at 6 h and 24 h, respectively. Overall, a higher number of genes were upregulated (1249 at 6 h, 1028 at 24 h) compared to downregulated (553 at 6 h, 735 at 24 h) in response to NTHi.

To assess regulation of the 1802 and 1763 DEGs at each time point, the 6 h and 24 h DEG lists were compared for conserved genes. This comparison identified 863 DEGs (hereafter designated as ‘core DEGs’) to be differentially expressed between uninfected and infected MDM at both 6 h and 24 h (**Figure 2C**). The remaining 1839 DEGs were differentially expressed at a single time point only, with 939 DEGs only differentially expressed at 6 h and 900 DEGs only differentially expressed at 24 h between uninfected and infected MDM.

The clustering profiles of each DEG group visualised with heatmaps, demonstrated time-dependent expression differences between the three groups. For the 6 h only DEGs, all 6 h infected samples clustered together, however they also clustered independently of the 24 h infected samples (**Figure 2D**). Similarly, for the 24 h only DEGs, the 24 h infected samples clustered together but away from the 6 h infected samples and the uninfected samples (**Figure 2E**). In contrast, for the 863 core DEGs, all infected samples clustered independently of the uninfected samples, but clustered together regardless of time point (**Figure 2F**). The absence of a strong time point signal within this core DEG list was perhaps due to sustained gene expression across time points, emphasised by only 9 out of the 863 genes changing direction of expression between 6 h and 24 h (**Table S1**).

Gene list enrichment analysis found only a small number of significantly enriched GO:terms within the 6 h only DEG list for all three GO categories. Only 6 terms (1 Biological Process and 5 Cellular Component) were determined to be significantly enriched (FDR p<0.05), with no enrichment in the Molecular Function GO category. In contrast, significantly enriched terms were identified for all three GO categories for the 24 h only DEGs (**Table 1**). Finally, significant enrichment of numerous immune response terms in the GO:Biological Process category was identified in the core DEG list (**Table 1**).

**Table 1:** Time-dependent enrichment of macrophage processes during NTHi persistence.

**Category**	**GO:ID**	**GO:Term**	**Enrichment FDR**	**Genes in input**	**Genes in annotation**
6h only DEGs					
GO: Biological Process	GO:0001816	cytokine production	0.05	71	875
GO: Cellular Component	GO:0098589	membrane region	0.03	39	411
	GO:0045121	membrane raft	0.03	37	396
	GO:0098857	membrane microdomain	0.03	37	397
	GO:0016324	apical plasma membrane	0.04	35	373
	GO:0000790	nuclear chromatin	0.05	121	1923
GO: Molecular Function	NA	NA	NA	NA	NA
24h only DEGs					
GO: Biological Process	GO:0022610	biological adhesion	1.93E-05	116	1516
	GO:0042493	response to drug	1.93E-05	97	1194
	GO:0007155	cell adhesion	1.93E-05	115	1509
	GO:0046903	secretion	2.88E-05	132	1835
	GO:0006952	defense response	3.85E-05	132	1850
GO: Cellular Component	GO:0031226	intrinsic component of plasma membrane	4.87E-17	161	1790
	GO:0005887	integral component of plasma membrane	4.87E-17	156	1710
	GO:0031012	extracellular matrix	1.21E-04	55	598
	GO:0098797	plasma membrane protein complex	1.83E-04	61	711
	GO:0062023	collagen-containing extracellular matrix	1.83E-04	46	474
GO: Molecular Function	GO:0005178	integrin binding	1.36E-03	23	146
	GO:0015267	channel activity	1.36E-03	62	705
	GO:0022803	passive transmembrane transporter activity	1.36E-03	62	706
	GO:0022836	gated channel activity	1.36E-03	48	491
	GO:0022843	voltage-gated cation channel activity	1.73E-03	30	245
6h24h ‘core’ DEGs					
GO: Biological Process	GO:0006952	defense response	4.82E-51	228	1850
	GO:0034097	response to cytokine	1.51E-48	183	1287
	GO:0043207	response to external biotic stimulus	1.17E-44	200	1606
	GO:0051707	response to other organism	2.36E-44	199	1604
	GO:0019221	cytokine-mediated signaling pathway	2.36E-44	138	814
GO: Cellular Component	GO:0005887	integral component of plasma membrane	5.91E-07	123	1710
	GO:0031226	intrinsic component of plasma membrane	5.91E-07	127	1790
	GO:0009986	cell surface	1.51E-06	84	1036
	GO:0031012	extracellular matrix	6.59E-03	48	598
	GO:0065010	extracellular membrane-bounded organelle	1.09E-02	5	9
GO: Molecular Function	GO:0005125	cytokine activity	3.92E-32	65	225
	GO:0048018	receptor ligand activity	7.37E-23	82	497
	GO:0005126	cytokine receptor binding	7.40E-23	64	312
	GO:0030546	signaling receptor activator activity	7.40E-23	82	502
	GO:0030545	receptor regulator activity	2.10E-20	82	547

A maximum of 5 of the most significantly functionally enriched terms for each category are shown, with fewer terms meaning lower enrichment significance for a specific category or time point. Genes in input show the number of MDM genes assigned to each term, which were compared against the full gene list for each category used by ToppFunn/ToppGene.

### Enrichment of Macrophage Immune Responses During NTHi Persistence

**Figure 3:**
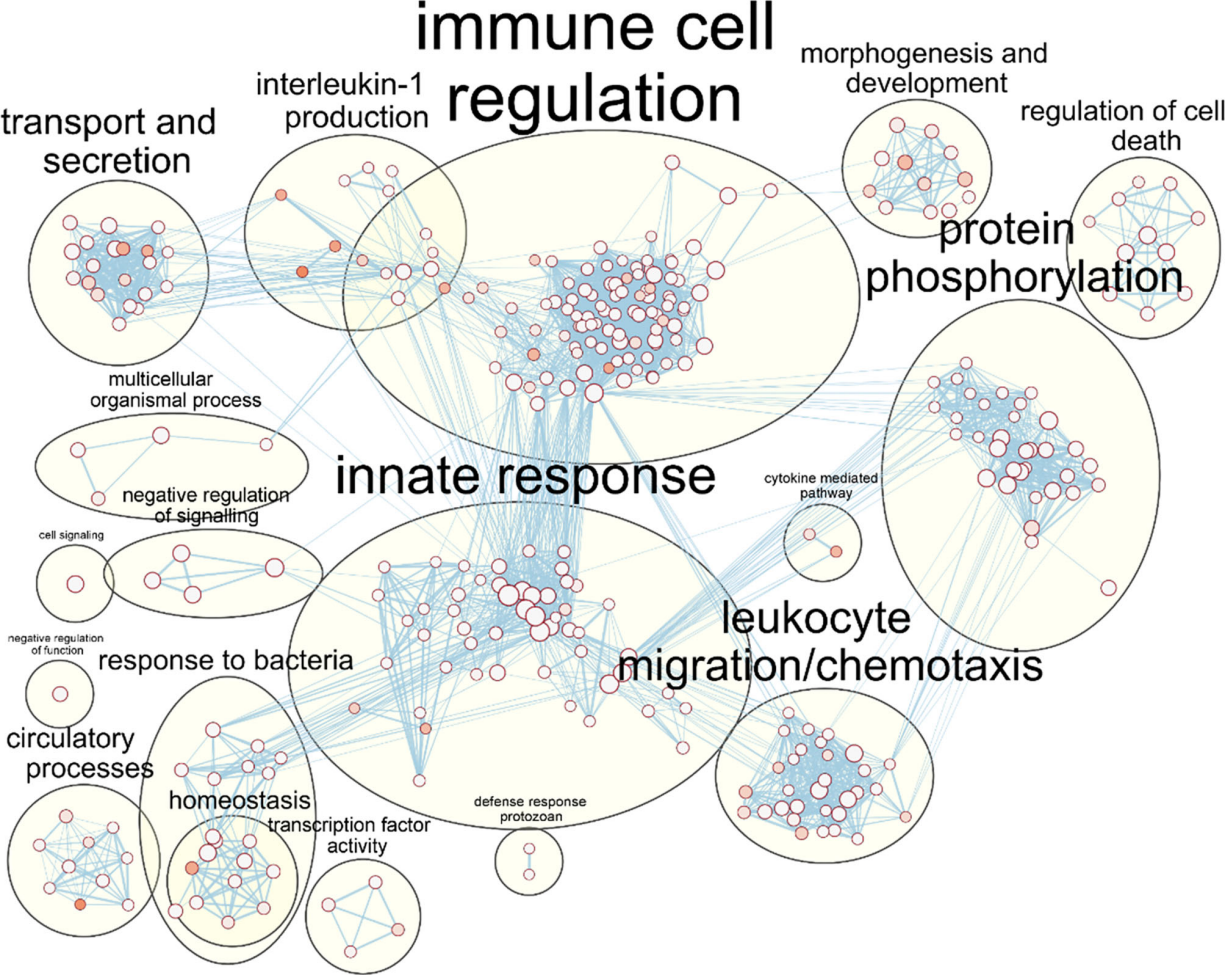
Enrichment of macrophage immune responses during NTHi persistence. Enrichment analysis using ToppFunn identified over 500 significantly enriched biological processes which were clustered using EnrichmentMap and AutoAnnotate in Cytoscape to identify the key biological processes involved in the MDM response to NTHi. Nodes represent individual GO:terms, with size relating to the number of genes in each term and the colour indicating enrichment significance. Edges represent connections between nodes that share genes.

As the core DEG list was significantly functionally enriched in immune processes, this core DEG list was explored further. To facilitate easier interpretation of functional enrichment, a network of the top 500 significantly enriched Biological Process terms was created using EnrichmentMap (Merico et al., 2010). The network further highlighted the immune response signal in the data, with gene terms clustering together under immune response phrases such as ‘immune cell regulation’, ‘innate response’, ‘response to bacteria’ and ‘leukocyte chemotaxis/migration’ (**Figure 3**).

Validation of MDM genes by qPCR confirmed activation of macrophage immune responses. Expression of *RELA* (p65 subunit for the transcription factor NF-κB) and *ACOD1* (Aconitate Decarboxylase 1) was significantly increased at both 6 h (2.4 FC for *RELA* and 179 FC for *ACOD1*) and 24 h (2.7 FC for *RELA* and 2759 FC for *ACOD1*) compared to the uninfected macrophage controls (all p<0.05, **Figures S4A, B**).

Protein level validation further demonstrated activation of macrophage pro-inflammatory responses during NTHi persistence, with increased levels of IL-1β, IL-6, TNF-α, and IL-10 released into culture supernatants detected in all infected samples compared to uninfected samples at each time point (all p<0.05, **Figures S4C–F**). Increased MDM release of neutrophil (IL-8, IL-17C) and lymphocyte (IL-15, IL-36β) related mediators in cell culture supernatants was also observed (**Figures S4G–J**).

### Enrichment of Macrophage Intracellular Immune Response Pathways Indicates Intracellular Residence of NTHi

**Figure 4:**
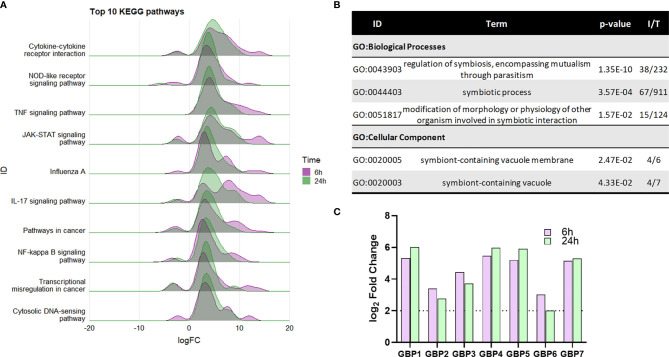
Enrichment of macrophage intracellular immune responses during NTHi persistence. **(A)** KEGG pathway analysis identified a number of enriched immune processes, with a number of pathways indicating a response to an intracellular pathogen. The genes assigned to each process were more highly upregulated at both 6 h (purple) and 24 h (green). Pathway/category IDs are ordered by enrichment significance (FDR). **(B)** Significantly enriched GO terms in the Biological Process and Cellular Component categories relating to host-pathogen symbiosis for the 863 core genes differentially expressed at 6 h and 24 h (log_2_ FC ± 2, FDR p < 0.05). P-value indicates the enrichment FDR, I = input number of genes, T= total number of genes in annotation. **(C)** MDM upregulation of guanylate-binding proteins (GBPs) 1-7 involved in host response to intracellular pathogens. Purple bar = 6 h, green bar = 24 h. Dotted line indicates log_2_ FC2 cut off. All genes were statistically significantly upregulated at both time points (FDR p < 0.05).

A total of 75 KEGG pathways were significantly functionally enriched [ShinyGo (Ge et al., 2020), FDR p<0.05, hypergeometric test] in the core DEG list, which included a number of immune response pathways (**Figure 4A**). Significant enrichment of specific intracellular immune response pathways such as the ‘NOD-like receptor signalling’, ‘Influenza A’ and ‘Cytosolic DNA-sensing’ KEGG pathways indicate activation of macrophage intracellular responses during NTHi persistence.

Within the Cellular Component category, enrichment of the GO:term ‘symbiont-containing vacuole’ (GO:0020003, FDR p=0.0433, **Figure 4B**), also suggest intracellular responses to NTHi. The genes enriched in this category (*GBP2, GBP4, GBP6*, and *GBP7*) are members of the guanylate-binding protein (GBP) family, which play a role in antibacterial defence against intracellular pathogens. Although not included within this particular GO:term annotation, 3 other GBP family members (*GBP1, GBP3* and *GBP5*) were also differentially expressed at both 6 h and 24 h (all FDR p<0.05, **Figure 4C**).

### NTHi Transcriptomic Regulation During Adaptation to Intracellular Persistence

**Figure 5:**
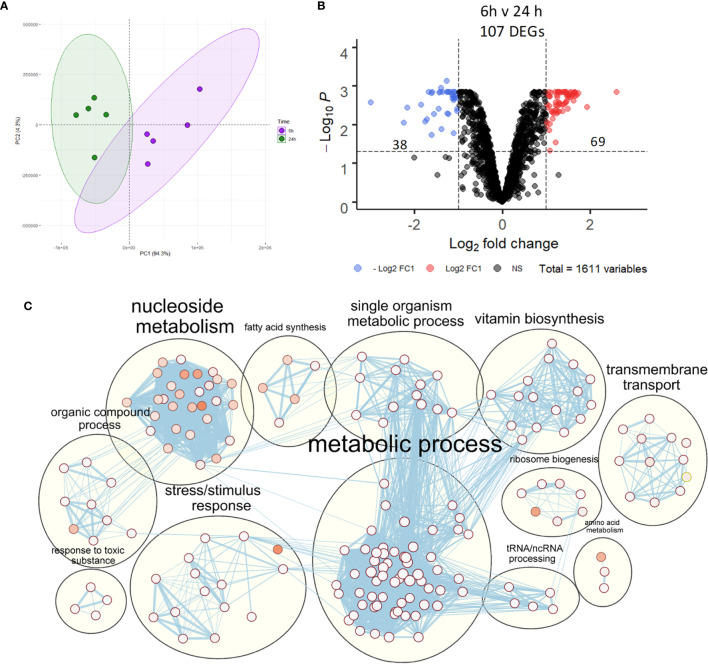
NTHi transcriptomic regulation during adaptation to intracellular persistence. **(A)** Principal component analysis identified clustering of NTHi samples based on time point (6 h time point samples in purple and 24 h time point samples in green). **(B)** Differential gene expression analysis identified 107 NTHi DEGs at 24 h (log_2_ FC1 cut off, FDR p < 0.05). **(C)** Clustering of the enriched Biological Process GO:terms performed using EnrichmentMap and AutoAnnotate in Cytoscape found enrichment of numerous metabolic processes. Nodes represent individual GO:terms, with size relating to the number of genes in each term and the colour indicating enrichment significance. Edges represent connections between nodes that share genes.

Despite activation of macrophage innate immune responses, NTHi was still able to persist within MDM for the duration of this model. Thus, the NTHi transcriptome was assessed to determine NTHi transcriptomic adaptations to intracellular persistence. PCA identified two distinct clusters separated by the first principal component (94.3%) which was associated with the 6 h or 24 h time point (**Figure 5A**). Differential gene expression analysis identified 107 DEGs between 6 h and 24 h, with 69 upregulated and 38 downregulated DEGs (**Figure 5B**, log_2_ FC ± 1, FDR p<0.05).

### NTHi Modulation of Metabolic Pathways During Intracellular Persistence

**Figure 6:**
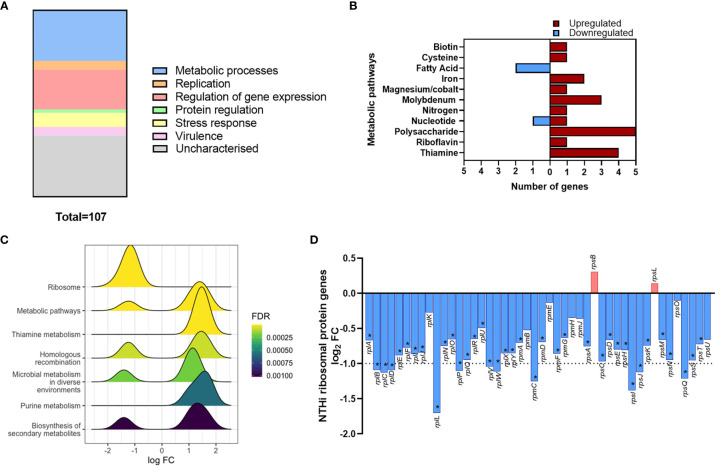
Modulation of NTHi processes during adaption to intracellular persistence. **(A)** The stacked bar chart highlights the main processes that the 108 NTHi DEGs are involved in. The process with the highest number of genes was metabolic processes (29), followed by regulation of gene expression (23), stress responses (8), virulence (5), replication (5) and protein regulation (2). The remaining genes (36) were uncharacterised (hypothetical, novel genes or sRNA). **(B)** Genes involved in bacterial metabolism were assigned to specific alternate metabolic pathways. Red = upregulated, blue = downregulated. **(C)** KEGG pathway analysis identified enrichment of KEGG pathways during intracellular persistence, ordered by enrichment significance (FDR). **(D)** KEGG pathway analysis identified the most significantly enriched pathway was ‘Ribosome’. Exploration of ribosomal protein gene expression identified global downregulation of NTHi ribosomal protein genes during infection. Bar chart shows log_2_ FC values of the 46 ribosomal protein genes detected in the NTHi data set. Dotted line indicates log_2_ FC1 cut off, with asterisk indicating genes (37) that were determined to be significantly differentially expressed at FDR p < 0.05.

Gene list enrichment analysis of the NTHi DEG list identified numerous significantly enriched terms in the Biological Process, Molecular Function and Cellular Component categories (**Figure S5**). Clustering the significantly enriched Biological Process terms identified that the majority of significantly enriched GO:term clusters were metabolic related, with ‘vitamin biosynthesis’, ‘amino acid metabolism’, ‘nucleoside metabolism’ and ‘fatty acid synthesis’ clusters identified (**Figure 5C**). Due to the high redundancy and ambiguity surrounding gene ontology terms, the functional role of the 107 DEGs was summarised using the results of the enrichment analysis and gene function (**Figure 6A**). The highest number of genes (29) were primarily involved in metabolic processes. The remaining genes were involved in regulation of gene expression (23), stress responses (8), virulence (5), replication (5) and protein regulation (2). The 35 remaining genes were uncharacterised, resulting in no gene name or function being available for analysis. Of these uncharacterised genes, 22 were hypothetical protein coding genes, 9 were transcripts assigned as novel genes and 2 were sRNA.

Out of the 107 DEGs, 29 metabolic-associated genes were identified, with upregulation of 5 genes (*rsxC, rsxD, rsxE, rsxG* and *cydD*) involved in aerobic respiration. The remaining genes, except *yhje_1*, were assigned to an alternative metabolic pathway, revealing a diverse array of metabolic pathways including biotin (vitamin B7), riboflavin (vitamin B2) and thiamine (vitamin B1) pathways (**Figure 6B**). Other annotated clusters demonstrated enrichment of bacterial stress responses (‘stress/stimulus response’ and ‘response to toxic substance’ clusters) and processes involved in gene expression and protein synthesis (‘ribosome biogenesis’ and ‘tRNA/ncRNA processing’ clusters).

Although KEGG pathway analysis confirmed enrichment of several metabolic KEGG pathways, the most significantly enriched KEGG pathway was the ‘Ribosome’ pathway (**Figure 6C**). All 11 DEGs assigned to this pathway were downregulated at 24 h. In total, 46 ribosomal protein genes were present in the annotated NTHi gene list, with 37 genes downregulated at 24 h (all FDR p<0.05, **Figure 6D**). However, only 11 of these 37 DEGs (*rplB, rplC, rplD, rplL, rplP, rplV, rplW, rpmC, rpsI, rpsJ* and *rpsQ*) were above the log_2_ FC1 cut off. Nonetheless, it was clear that between 6 h and 24 h, NTHi globally downregulated the expression of ribosomal protein genes.

### The Top Regulated NTHi Genes During Intracellular Persistence Were Differentially Expressed Compared to Planktonic NTHi

It was important to confirm that NTHi DEGs were only differentially expressed during intracellular persistence of MDM. Two significantly upregulated DEGs (*bioC* and *mepM*) and the top significantly downregulated DEG (*dps*) were selected for *in vitro* investigations. The expression of *bioC* (1.8 FC, p=0.0156), *mepM* (2.5 FC, p=0.0313) and *dps* (0.4 FC, p=0.0156) during NTHi persistence in MDM was first validated by qPCR (**Table S2**).

**Figure 7:**
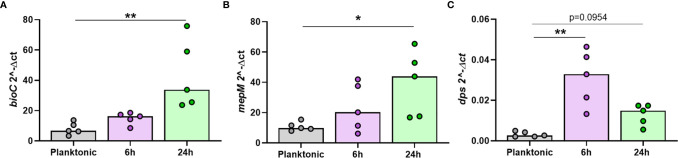
The top regulated NTHi genes during intracellular persistence were differentially expressed compared to planktonic NTHi. To compare gene expression between planktonic and intracellular, persisting NTHi, RNA was harvested from NTHi ST14 grown in culture media alone (regarded as planktonic NTHi) or from NTHi-infected MDM at the 6 h and 24 h time points, as previously described (n = 5). The expression of the top regulated NTHi genes **(A)** *bioC*, **(B)** *mepM* and **(C)** *dps* was assessed by qPCR. Gene expression was normalised to NTHi *rho* gene. Graphs show unpaired data and lines indicate medians. N = 5. Data were analysed using a Kruskal-Wallis test with Dunn’s multiple comparisons; *p < 0.05, **p < 0.01.

Next, the expression of each gene was compared between planktonic NTHi and intracellular NTHi at 6 h and 24 h. All three genes were more highly expressed by intracellular NTHi. The two upregulated DEGs *bioC* and *mepM* were more highly expressed at 24 h (p=0.0014 and p=0.0178, respectively, **Figures 7A, B**). The *dps* gene was more highly expressed by NTHi at 6 h compared to planktonic (p=0.0018), with expression levels decreasing by 24 h (p=0.0954), in line with the decrease in expression detected by dual RNASeq and qPCR validation between 6 h and 24 h (**Figure 7C**). Despite this decrease in expression, *dps* expression levels did not revert back to similar expression levels as planktonic NTHi, indicating the regulation of these genes during intracellular persistence were distinct compared to planktonic NTHi.

### Strain-Dependent Differences in NTHi Transcriptomic Adaptations During Intracellular Persistence

**Figure 8:**
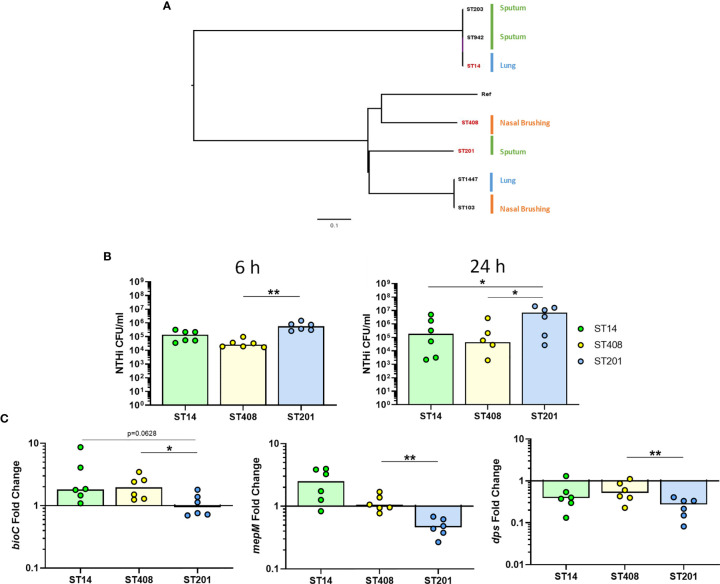
Strain-dependent differences during NTHi persistence. **(A)** The diversity of seven clinical NTHi isolates were assessed by ParSNP using default parameters and NTHi 86-028NP as the reference strain. Strains were isolated from either from sputum sample (green), nasal brushing (orange) or protected bronchial brushes of the lung (blue). Phylogenetic tree was created in FigTree using ParSNP output files and strains highlighted in red (ST14, ST408 and ST201) indicate the strains chosen for further *in vitro* experimental analysis. **(B)** Invasion and persistence within MDM by the three chosen different strains of NTHi was measured by live viable counting at the 6 h and 24 h time points. **(C)** Expression of the top regulated NTHi genes were differentially expressed by additional clinical strains of NTHi during intracellular persistence. Gene expression was normalised to NTHi *rho* gene and data are shown as fold change in expression from 6 h to 24 h Graphs show paired data and lines indicate medians. N = 6. Data were analysed using Friedman test with Dunn’s multiple comparisons; *p < 0.05, **p < 0.01.

As NTHi strains are heterogeneous and have been suggested to have different capacities to persist within host cells (Craig et al., 2001), we also assessed whether the expression of the top three identified NTHi genes were conserved in additional clinical strains during intracellular persistence of MDM at 24 h. The genomic relatedness of seven clinical strains of NTHi isolated from nasal brushes, lung protected brushes or sputum obtained from patients with chronic respiratory disease was assessed by ParSNP (Treangen et al., 2014) (**Figure 8A**). Three strains were selected based on the diversity inferred from the phylogenetic tree, including the ST14 strain, which has been used for this work so far. To ensure diverse strains were selected, one strain from each clade representing a different anatomical sampling location was chosen: ST408 (nasal brushing), ST14 (lung protected brushing), ST201 (sputum).

All three strains were able to persist within MDM (**Figure 8B**), however higher levels of ST201 were recovered from MDM at 6 h (p=0.0016 compared to ST408) and 24 h (p=0.0281 compared to both ST14 and ST408). When comparing the expression of each of the three genes across strains, it was apparent that NTHi ST201 modulated expression of the three genes more robustly than ST14 and ST408 during persistence (**Figure 8C**).

## Discussion

Rapid advances in sequencing technologies now allow for simultaneous profiling of host-pathogen interactions, giving novel insights into the cellular cross-talk occurring during clinically relevant infections. NTHi is still considered an extracellular pathogen, despite increasing evidence of an intracellular lifestyle (Craig et al., 2001; Morey et al., 2011; Olszewska-Sosińska et al., 2016). NTHi was first demonstrated to be residing and replicating within the phagocytic compartment of mononuclear cells (Farley et al., 1986), but in epithelial cells, NTHi colocalised with acidic compartments displaying late endosomal features and did not appear to be replicating (Morey et al., 2011). Subsequent *in vitro* studies have shown the ability of NTHi to invade and persist within monocytes and macrophages (Ahrén et al., 2001; Craig et al., 2001, 2002; King et al., 2008). The mechanism underlying this NTHi persistence within host cells is unclear. As such, we used dual RNASeq to simultaneously assess host and pathogen transcriptomic changes during intracellular persistence to better understand the mechanism of NTHi persistence within human macrophages.

Analysis of macrophage transcriptomic changes in response to NTHi persistence identified time-dependent macrophage responses. Specifically, a core transcriptomic profile was consistently expressed across both 6 h and 24 h, which was enriched in intracellular immune responses. A component of the macrophage intracellular pathogen detection machinery includes GBPs, of which all 7 GBPs were upregulated during NTHi persistence. The importance of GBPs in restricting intracellular pathogens have been shown in studies using GBP-deficient macrophages, which demonstrate impaired responses to intracellular pathogens including *Mycobacterium bovis*, *L. monocytogenes*, *Francisella novis* and *Salmonella typhimurium* (Kim et al., 2011; Meunier et al., 2014; Meunier et al., 2015). GBPs are thought to facilitate rupturing of either bacteria-containing vacuoles or bacteria present in the cytosol. Subsequent release of bacterial content results in cytosolic detection of the invading pathogen and inflammasome activation (Meunier et al., 2014; Meunier et al., 2015), which could explain macrophage enrichment of the ‘Cytosolic DNA-sensing’ pathway in response to NTHi.

Conversely, recent work has shown GBPs associate with the bacterial surface moments after bacterial escape from host vacuoles, without bacteriolysis (Santos et al., 2020). The second highest upregulated NTHi DEG was an endopeptidase, *mepM*, which could suggest GBP-NTHi interactions. Endopeptidases are responsible for incorporation of peptidoglycan into the bacterial cell wall, a crucial process not just for bacterial growth and replication, but also bacterial cell viability. Thus, upregulation of *mepM* could suggest activation of NTHi defences against host immune mechanisms that target the cell wall of bacteria. Functional work investigating physical NTHi-GBP interactions within MDM are therefore warranted to assess the role of GBPs in the response to intracellular NTHi.

We identified *ACOD1* as one of the top most significantly upregulated MDM genes across both 6 h and 24 h. *ACOD1* encodes for a cis-aconitate decarboxylase involved in the production of itaconate from cis-aconitate produced in the TCA (Kreb) cycle (Michelucci et al., 2013). Itaconate inhibits succinate dehydrogenase (SDH), resulting in accumulation of succinate and diversion of macrophage metabolism towards aerobic glycolysis (O’Neill and Artyomov, 2019). *ACOD1* was previously designated *IRG1* (immune responsive gene 1) as it appeared to play an unknown function in the inflammatory immune response, with increased gene expression measured in LPS-stimulated macrophages (Lee et al., 1995). *ACOD1* and itaconate are suggested to be immunomodulatory, with studies demonstrating that *ACOD1* expression was important for the host immune response to *Mycobacterium tuberculosis* (Mtb) infection (Nair et al., 2018; Hoffmann et al., 2019). However, Michelucci et al., confirmed that itaconate also functions as an antimicrobial metabolite, important for restricting growth of Mtb and *Salmonella enterica* (Michelucci et al., 2013).

In contrast, some pathogens can utilise itaconate to enhance pathogenicity. Riquelme et al. found that in response to itaconate, *Pseudomonas aeruginosa* adapted metabolic activity towards biofilm formation and extracellular polysaccharide (EPS) production, which in turn resulted in increased itaconate release from host cells (Riquelme et al., 2020). Similarly, *Salmonella* has shown to be able to sense and respond to macrophage itaconate by upregulating expression of itaconate degradation proteins (Hersch and Navarre, 2020). It is not known whether NTHi is similarly able to interfere with *ACOD1*/itaconate regulation of macrophage inflammatory processes, however the presence of genes for itaconate degradation in numerous other bacteria suggests this possibility (Sasikaran et al., 2014). Further work assessing the exact impact of the antibacterial and immunomodulatory activity of itaconate during NTHi persistence will help identify whether these properties of itaconate can feasibly be used therapeutically to reduce the burden of NTHi persistence in chronic respiratory diseases. This is particularly important given recent findings that airway itaconate levels and expression levels of *ACOD1* were decreased in AM from patients with IPF compared to controls (Ogger et al., 2020).

A crucial macrophage function is to orchestrate and regulate the immune response, which includes recruitment and activation of other immune cells (Arango Duque and Descoteaux, 2014). NTHi persistence has been specifically associated with a switch to T17 and neutrophilic inflammation in asthma (Yang et al., 2018). In this current work, KEGG analysis identified enrichment of IL-17 signalling, with upregulation of numerous macrophage genes for neutrophil and T cell chemoattractants present in the ‘leukocyte chemotaxis/migration’ biological process cluster. Macrophage release of specific neutrophil-associated mediators (IL-8 and IL-17C) was detected in cell culture supernatants. NTHi-infected macrophages could be a cellular source of neutrophil chemoattractants *in vivo*, driving the recruitment of neutrophils to the lung. This has previously been postulated by Song et al., who suggested that alveolar macrophages are the cellular source of increased IL-17 in the BALF of asthmatic patients, not Th17 cells (Song et al., 2008).

In contrast, Singhania et al., suggested that activated T cells drive an IL-17 response in severe asthma (Singhania et al., 2018). We have previously demonstrated the ability of macrophages to activate T cells in response to challenge with influenza A (Staples et al., 2015) or NTHi (Wallington et al., 2018). In particular, a role for the PD1/PDL1 exhaustion pathway in regulating T cell function has been suggested to contribute to dysfunctional cytotoxic responses and impaired immune regulation in an experimental lung explant model (McKendry et al., 2016). Given the vital role of the macrophage in immune regulation, the complex, inflammatory environment in asthma may be driven by dysregulation of the NTHi-infected macrophages ability to recruit and activate both neutrophils and T cells.

Intracellular invasion and persistence within host cells is a strategy employed by numerous bacteria to evade the immune response. As described previously, invasion of macrophages by NTHi has been reported by numerous studies (Ahrén et al., 2001; Craig et al., 2001; Craig et al., 2002; King et al., 2008; Morey et al., 2011), but the mechanism of persistence and survival of NTHi within macrophages is unclear. Following invasion of host cells, bacteria are faced with a hostile environment which is only available as a niche for those bacteria able to adapt to unfavourable conditions. Macrophage activation of responses are crucial for killing intracellular pathogens, with some pathogens such as Mtb able to persist within macrophages that have become activated, whereas other pathogens, such as *Listeria monocytogenes* are more readily killed following activation of macrophage intracellular immune responses (Kaufmann and Dorhoi, 2016). Thus, bacterial adaptations to a hostile intracellular environment can determine the ability of pathogens to persist. Adaptations to environmental changes, such as nutrient or oxygen availability, can be regulated by the bacterial stringent response (Wilson and Nierhaus, 2007). Differential regulation of NTHi genes involved in various metabolic pathways, stress responses and the ribosome pathway, as well as no change in NTHi CFU between 6 h and 24 h, suggested potential activation of the bacterial stringent response by NTHi to enhance intracellular survival.

Global modulation of the ribosome pathway has been observed in other pathogens during intracellular infection of macrophages, including *Bordetella pertussis* and *Leishmania* (Dillon et al., 2015; Petráčková et al., 2020). As well as a component of the stringent response, ribosomes are targets of antibiotics, so it is possible that downregulation of ribosome biogenesis may be a mechanism of bacterial defence against antibiotics. One such antibiotic which targets the bacterial ribosome is the macrolide azithromycin. A study by Taylor et al. demonstrated that long term azithromycin treatment reduced *H. influenzae* load and exacerbation risk in severe asthmatics (Taylor et al., 2019). However, this reduction was associated with increased carriage of antibiotic resistance genes by certain bacteria. Furthermore, Olszewska-Sosińska et al., found that persistent NTHi isolates recovered from macrophages obtained from azithromycin-treated children were not azithromycin resistant (Olszewska-Sosińska et al., 2016). As such, rather than active resistance to antibiotics conferred by specific antibiotic resistance genes, the maintenance of persister cells and drug tolerance may occur by other mechanisms, such as downregulation of ribosomes during intracellular persistence. These findings highlight the crucial importance of rapidly identifying and developing novel antimicrobials, given the global antimicrobial resistance crisis. The data set generated from this work provides a rich resource for exploration or screening of bacterial genes that may be associated with intracellular survival which may identify novel targets for antimicrobial therapeutics.

This work identified significant enrichment of various bacterial metabolic pathways, in agreement with a previous pioneering dual RNASeq study investigating NTHi-epithelial cell interactions (Baddal et al., 2015). Metabolic adaptations to changes in host substrate availability have been suggested to contribute to NTHi pathogenesis (Othman et al., 2014). In this current work, the *bioC* gene displayed the highest level of upregulation in the NTHi data set and was significantly upregulated compared to planktonic NTHi. Encoding for an O-methyltransferase, *bioC* is involved in generating the pimeloyl acyl carrier protein (ACP) by the fatty acid synthesis pathway and can be used as a precursor for biotin synthesis (also known as vitamin H or B7) (Lin and Cronan, 2012). Biotin is a limited intracellular resource, thus intracellular pathogens able to scavenge or generate biotin in biotin-restricted environments could be better adapted to survive in an intracellular niche. This concept is supported by studies demonstrating the importance of biotin for survival and fitness of other intracellular pathogens (Yu et al., 2011; Napier et al., 2012; Sprenger et al., 2020). As biotin synthesis pathways are absent in humans, components of the biotin synthesis pathway could be attractive targets for therapeutics against intracellular pathogens. Determining whether NTHi intracellular persistence is dependent on the ability of NTHi to scavenge host biotin could identify biotin synthesis pathways as a potential therapeutic target.

In the current dataset, several genes previously reported to be important for NTHi oxidative stress responses (Harrison et al., 2007) were downregulated or not differentially expressed. This could suggest NTHi escape from macrophage intracellular killing mechanisms by 24 h. In particular, the *dps* gene has been suggested to play a role in protecting NTHi from oxidative stress and has also been identified to be crucial for NTHi biofilm growth (Pang et al., 2012; Swords, 2012). The *dps* gene exhibited the highest decrease in expression at 24 h, suggesting NTHi adaptation to intracellular residence within MDM is distinct to that of biofilm growth. Differences between NTHi lifestyles could have important implications for development of effective therapeutics. Although this work attempted to validate the results of the dual RNASeq transcriptomic analysis using planktonic NTHi by qPCR, one of the main limitations of this analysis is the absence of sequenced planktonic comparisons. Sequencing planktonic NTHi would have allowed for assessment of the differences in the transcriptomic profiles between planktonic and intracellular NTHi.

Additionally, as well as the previously discussed antibacterial functions, azithromycin also exhibits immunomodulatory functions, including antioxidant properties (Bergamini et al., 2009; Mal et al., 2013). However, dampening of oxidative stress responses in the airway may in fact promote NTHi persistence. NTHi utilises multiple strategies to promote a multifaceted defence response against oxidative stress, including regulation of dps (Harrison et al., 2012). As *dps* was downregulated at 24 h compared to 6 h in all three strains tested in this current work, it is possible that multiple strains of NTHi can overcome host oxidative stress responses to facilitate intracellular persistence. Thus, azithromycin-mediated dampening of antioxidant responses may contribute to NTHi persistence in the airway. Instead, the other immunomodulatory functions of azithromycin may be more important in reducing NTHi load, including increasing macrophage phagocytosis, upregulating expression of cell surface receptors (e.g. mannose receptor) and reducing pro-inflammatory cytokine levels (Hodge et al., 2006; Hodge et al., 2008; Euba et al., 2015). The AMAZES trial showed that despite a significant decrease in *H. influenzae* copy number, carriage of NTHi was still evident in some patients (Taylor et al., 2019). Thus, strain-dependent differences in NTHi adaptation to the lung environment may be responsible for the chronic airway colonisation by some strains; determining the transcriptomic profile of multiple diverse NTHi strains persisting despite azithromycin treatment would be informative.

NTHi strains are heterogeneous, with strain-dependent differences in macrophage persistence shown in this current work and that of Craig *et al*. (Craig et al., 2001). The seven strains of NTHi used for phylogenetic analysis were all clinical isolates cultured from either protected bronchial brushings, induced sputum or nasal brushings. However, strains did not cluster according to sample type. Although this is a small sample size, dissimilarity of NTHi strains derived from the same clinical source has also been shown in two larger studies (Erwin et al., 2008; De Chiara et al., 2014). Furthermore, two invasive NTHi strains (C188 and R2866) displayed increased ability to metabolise diverse substrates compared to a COPD isolate (Hi2019), whereas Hi2019 was better able to invade and reside within airway cells (Muda et al., 2019). This comparative analysis suggests differences in metabolic adaptations could underpin the ability of NTHi to persist in certain anatomical niches, further supported by the different levels of gene expression exhibited by the three diverse strains used in this current study. Thus, stratifying NTHi strains by transcriptomic adaptations during persistence could be a better method by which to identify strains better able to persist *in vivo*. However, a limitation of our model is that it is likely too acute to consider the long-term implications of NTHi colonisation and associated transcriptomic changes in the airway. Future investigations assessing the key gene targets identified in this analysis using macrophages isolated from individuals chronically colonised with NTHi will confirm the significance of transcriptomic adaptations to NTHi intracellular persistence within macrophages in the airway.

We recognise another limitation of our current work was the use of MDM to model lung macrophages. Although MDM have been extensively used and display similar phenotypes and responses to alveolar macrophages (Akagawa et al., 2006; Tudhope et al., 2008; Taylor et al., 2010), they are likely to not be completely reflective of NTHi-macrophage interactions in the airway, particularly given the reported functional impairments of macrophages from individuals with chronic respiratory disease (Taylor et al., 2010; Staples et al., 2012; Berenson et al., 2013; Liang et al., 2014; Belchamber et al., 2019). Moreover, lung macrophages in COPD consist of various subpopulations, which also differ from control lung macrophages in terms of pro-inflammatory and phagocytic ability (Dewhurst et al., 2017). As such, it is possible that a specific sub-population of macrophages may function as a preferred niche for intracellular persistence. Notably, dual RNASeq analysis comparing responses of alveolar and interstitial macrophages (AM and IM, respectively) to infection with Mtb found that Mtb growth was more restricted in IM, which demonstrated a more pro-inflammatory response than AM (Pisu et al., 2020). Thus, it would be important to confirm the transcriptomic findings in this study using lung macrophages from chronic respiratory disease patients colonised with NTHi.

## Final Conclusions

In summary, this work demonstrates that despite significant enrichment of immune processes and intracellular pathways by MDM, NTHi was able to persist for the duration of our experiments. Thus, macrophages may act as a protected niche within the airway, promoting NTHi colonisation, especially in chronic respiratory diseases that already have evidence of macrophage dysfunction. Furthermore, this persistence may contribute to the dysregulated immune response in chronic respiratory disease, as upregulation of macrophage pro-inflammatory responses, including mediators of other inflammatory immune cells, were detected in response to NTHi persistence. Analysis of NTHi gene expression during persistence revealed metabolic adaptations which may be crucial for bacterial persistence. However, further functional work is needed to confirm the importance of genes and pathways before they are deemed key targets to be taken forward into drug development pipelines for validation. Nonetheless, this work provides a rich transcriptomic resource for exploration of host-pathogen interactions, which could unveil novel gene targets for therapeutic interventions to reduce the burden of NTHi in the airway.

## Data Availability Statement

The original contributions presented in the study are publicly available. This data can be found here: GSE180166 (http://ncbi.nlm.nih.gov.geo/).

## Ethics Statement

The studies involving human participants were reviewed and approved by Hampshire A Research Ethics Committee (13/SC/0416). The patients/participants provided their written informed consent to participate in this study.

## Author Contributions

JA and KS conceptualized the project. JA, AH, DC, MC and KS contributed to methodology. JA and AH undertook the formal analysis. KS administered the project. JA performed the investigation. DC, MC, TW and KS provided resources and acquired funding. DC, MC, TW and KS supervised the project. JA, AH and DC curated the data. JA and KS wrote the original draft. All authors contributed to the article and approved the submitted version.

## Funding

This work was primarily funded by an Asthma UK studentship award (AUK-PHD-2016-363). We also gratefully acknowledge the support of the Southampton AAIR charity in funding this work. The funders had no role in study design, data collection and interpretation, or the decision to submit the work for publication.

## Conflict of Interest

DC reports that he was a post-doctoral researcher on projects funded by Pfizer and GSK between April 2014 and October 2017. TW reports grants and personal fees from AstraZeneca, personal fees and other from MMH, grants and personal fees from GSK, personal fees from BI, and grants and personal fees from Synairgen, outside the submitted work. KS reports grants from AstraZeneca, outside the submitted work.

The remaining authors declare that the research was conducted in the absence of any commercial or financial relationships that could be construed as a potential conflict of interest.

## Publisher’s Note

All claims expressed in this article are solely those of the authors and do not necessarily represent those of their affiliated organizations, or those of the publisher, the editors and the reviewers. Any product that may be evaluated in this article, or claim that may be made by its manufacturer, is not guaranteed or endorsed by the publisher.
