# Supplementary material for: Dual RNASeq Reveals NTHi-Macrophage Transcriptomic Changes During Intracellular Persistence
Source: Front Cell Infect Microbiol. 2021 Aug 23;11:723481. doi: 10.3389/fcimb.2021.723481 (PMC8419319; doi:10.3389/fcimb.2021.723481)
Supplement: Supplementary file 1 [file DataSheet_1.docx]

Supplementary Material

# Supplementary Figures

Total:
Figures = 5
Tables = 4

**Figure S1. Time course of intracellular NTHi levels**. MDM were infected with NTHi ST14 for 2 h, 6 h or 24 h followed by a 90 min gentamicin to wash remove extracellular NTHi in order to only recover intracellular NTHi. Each point represents the median of 4 experiments and lower and upper whiskers indicate IQR. Data were analysed by Friedman test with Dunn’s multiple comparison test; *p<0.05 for 6 h v 24 h comparison.

**Figure S2. Use of GFP-NTHi to visualise NTHi infection of macrophages**. **(A)** GFP-NTHi infected MDM were harvested at the 6 h and 24 h time point, lysed and plated to quantify the amount of GFP-NTHi associated with MDM. GFP-NTHi infected MDM were also analysed by flow cytometry, with the uninfected MDM acting as the negative gating control**. (B)** Histogram plot showing uninfected MDM (grey) 6 h GFP-infected MDM (purple) and 24 h GFP-infected MDM (green). **(C)** Quantification of MDM infection by GFP-NTHi, n=2. Bar shows median and lines indicate interquartile range. Graphs show paired data and lines indicate medians. Data were analysed by Wilcoxon signed-rank test and no statistical significance was determined.

**Figure S3. Percentage of mapped sequenced reads to host and pathogen reference genomes.** Mapping of the raw data to the each reference genome was performed separately by Novogene, with only clean reads used for mapping.

**Figure S4. Validation of MDM upregulation of innate immune responses.** MDM were infected as previously described and RNA or cell culture supernatants were harvested at the 6 h and 24 h time points. Macrophage gene expression of **(A)** *RELA* and **(B)** *ACOD1* was measured by qPCR and expression values were normalised to *B2M* and is expressed as deltact. Macrophage release of **(C)** IL-1β, **(D)** IL-6, **(E)** TNF-α **(F)** IL-10, **(G)** IL-8, **(H)** IL-15**, (I)** IL-17C and **(J)** IL-36β into cell culture supernatants was measured either by ELISA (IL-1β, IL-6, IL-8) or Luminex (TNF-α, IL-10, IL-15, IL-17C, IL-36β). Graphs show paired data and bars indicate median values. Data were analysed by Wilcoxon signed rank test; *p<0.05.

**Figure S5. Gene ontology analysis of the 107 NTHi differentially expressed genes**. Bubble plot visualisation of the top significant functionally enriched GO terms for the categories Molecular Function, Biological Process and Cellular component categories. The yellow line indicates 0.05 FDR enrichment p-value, size of dots indicate number of genes present in each term. The accompanying table shows the GO:terms indicated in the bubble plot, but for clarity, the GO:ID numbers are accompanied with the GO:terms and enrichment FDR value. A maximum of 5 of the most significantly functionally enriched pathways are shown. Input genes show the number of NTHi genes assigned to each term

# Supplementary Tables

**Table S1. Table of the 9 genes which change direction of expression during NTHi persistence.** Within the conserved ‘core’ macrophage gene set, only 9 out of 863 genes changed direction of expression during NTHi persistence. Values shown are log_2_FC between uninfected and infected MDM at the respective time points, all FDR p<0.05.

**Table S2. Top regulated NTHi gene function and confirmation of differential expression in MDM by qPCR.** Table shows the gene name of the top three regulated NTHi genes and the putative function of the gene product. The expression of these three genes was validated by qPCR using NTHi ST14 (original strain used for the sequencing analysis). Gene expression was normalised to NTHi *rho* gene and data are shown as fold change in expression from 6 h to 24 h. Data were analysed by Wilcoxon’s signed rank test, significant p-values are indicated in bold.

# Supplementary Methods

## Flow Cytometry analysis of GFP-NTHi infection of MDM

MDM were infected with GFP-NTHi as described and harvested at 6 h and 24 h using 200 µl non-enzymatic cell dissociation solution (Sigma) for 20 min at 37°C. After 20 min, cells were gently removed from wells into 5 ml polypropylene tubes (BD). Cells were washed and centrifuged at 400 g, 4°C for 5 min in Fluorescence-Activated Cell Sorting (FACS) buffer (2mM EDTA, 0.5% (w/v) BSA in PBS) and resuspended in 200 µl Cytofix/Cytoperm™ Fixation and Permeabilization Solution (BD Biosciences) and incubated on ice for 20 min. Cells were again washed and resuspended in 250 µl FACS buffer prior to acquisition using 9-colour FACSAria (BD Biosciences) and data were analysed by FlowJo software (Version 10).

## Quantification of mediator release by Luminex

Analysis of MDM cell culture supernatants for quantification of mediator release also performed using a customised 14-plex Luminex Human Magnetic Assay according to the manufacturer’s instructions (R&D Systems). Briefly, 50 µl of the premixed microparticle cocktail was added to each well of a 96 well plate and incubated with 50 µl of neat MDM culture supernatant or the pre-prepared standard at 4°C overnight. The plate was then washed with 100 µl Wash Buffer (R&D Systems) whilst attached to a Handheld Magnetic Washer, which held the microparticles and bound sample analytes to the bottom of the plate to prevent them washing away. The biotin antibody cocktail provided was diluted with the provided diluent concentrate (R&D Systems) and 50 µl was added to each well and incubated for 1 h on a plate shaker. The plate was again washed as described above, before 50 µl of Streptavidin-PE was added and again incubated for 30 min on a plate shaker. The plate was read using a Bio-Plex 200 system (Bio-Rad), with the concentration of each analyte calculated from the concentration of the 1 in 3 diluted standard curve for each analyte. The lower and upper limit of quantitation (LLOQ and ULOQ) for each analyte is indicated in Table S4.

## Supplementary Methods Tables

**Table S3.** List of TaqMan gene expression assays (all from Thermo Fisher) used for validation of MDM and NTHi gene expression by qPCR


**Table S4.** The lower and upper limit of quantitation (LLOQ and ULOQ) for each analyte (pg/ml) measured by a customised Luminex Human Magnetic Assay.
